# Retrolental fibroplasias: What lies beneath?

**DOI:** 10.3205/oc000141

**Published:** 2020-03-18

**Authors:** Parveen Sen, Dhaivat Shah

**Affiliations:** 1 Shri Bhagwan Mahavir Vitreoretinal Services, Medical Research Foundation, Chennai, Tamil Nadu, India

**Keywords:** retrolental fibroplasias, myelinated nerve fibers, masquerades

## Abstract

A nine-month-old female baby with normal birth history presented with her mother complaining of a white spot in the baby’s right eye, which the mother had noticed at five months of age. External photograph showed a retrolental fibroplastic membrane visible in the superior half of the dilated pupil. Retcam fundus photo revealed myelinated nerve fibers extending from the disc till the ora superiorly and forming a membranous fold. Intraoperative OCT confirmed thickened RNFL with compact retina. Thus, the retrolental fibroplasia turned out to be a masquerade for myelinated nerve fibers. Since it was not involving the visual axis with no coexisting traction, the mother was reassured regarding the benign nature of the condition.

## Case description

A nine-month-old female baby from rural North India born at 37 weeks with a birth weight of 2.1 kg presented with her mother complaining of a white spot in the baby’s right eye. The mother had accidentally noticed the spot when the child was around five months of age and she was referred to us by her local ophthalmologist with the query to rule out retinoblastoma. The child had central, steady, and maintained fixation in both eyes. Retinoscopy revealed a dull glow in the right eye with a cycloplegic refraction of –18.00 D and a clear red glow in the left eye with cycloplegic refraction of +1.00 D in undilated state. On torch light, leukocoria with normal anterior segment and clear lens was noted. The white reflex was more prominent from the superior one third of the pupil, more evident in the dilated state. Keeping in mind the differential diagnosis of retinoblastoma, retinopathy of prematurity and exudative retinopathy, an examination under anesthesia was advised.

**Figure 1:**
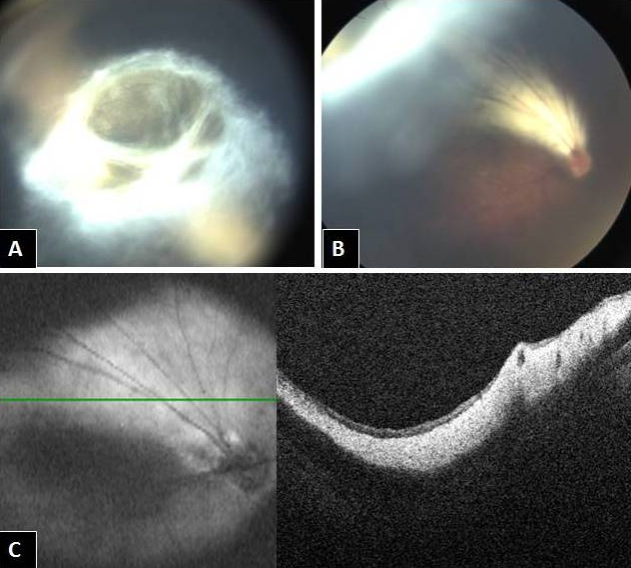
A) & B) Fundus photos showing a yellowish white shiny area superior to the disc extending till the superior ora and forming a membranous fold behind the lens in this area, giving an appearance of retrolental fibroplasias. C) Intraoperative OCT showing thickened RNFL with compact retina, which confirms the diagnosis of myelinated nerve fibers.

On examination under anesthesia, the fundus showed a yellowish white shiny area (Figure 1A (Fig. 1)) superior to the disc extending till the superior ora and forming a membranous fold behind the lens in this area, giving an appearance of retrolental fibroplasia (Figure 1B (Fig. 1)). Intraoperative OCT (Figure 1C (Fig. 1)) confirmed a thickened retinal nerve fiber layer (RNFL) with compact retina, which confirmed our diagnosis of myelinated nerve fibers (MNF).

## Discussion

Myelinated retinal nerve fiber is a congenital benign condition which is commonly found in conjunction with the optic nerve head and uncommonly noted in isolation over the fundus. It appears as a whitish or yellowish membrane over the retinal surface following the distribution of ganglion cell axons, giving it a frayed appearance [1].

The normal myelination process begins at around eight months in embryonic period and ends before birth [1].

Various hypotheses have been postulated, out of which the most commonly accepted one is the inhibition of oligodendrocyte migration due to cellular factors released from astrocytes just before birth [2], [3]. If this does not occur, the myelination continues beyond the lamina cribrosa around the optic nerve hypoplasia (ONH). In most cases, MNF is localized, but if the process continues, it can be as extensive as till the equator or beyond [1].

In our case, the MNF was extending from the ONH till the superior ora serrata and presumably might have formed a membranous fold at that site. This subsequently gave an appearance of retrolental fibroplastic membrane and showed a very typical white reflex on torch light examination. Taking the age and history into consideration, congenital cataract, retinoblastoma, retinopathy of prematurity or exudative retinopathy would be the primary suspects. A dilated fundus examination with thorough peripheral fundus evaluation and photo documentation is imperative in such cases, preferably under anesthesia. To add to our armamentarium of investigations, an intraoperative OCT was done and the thickened RNFL seen in the area of MNF was captured, which ascertained our diagnosis of MNF [4].

Since the retrolental membranous fold was fortunately neither involving the visual axis nor causing any concurrent traction, the observation of the same was advised and the mother was henceforth reassured regarding the benign nature of the condition. Regular use of glasses with amblyopia therapy was advised. A six-monthly follow-up was recommended.

## Conclusion

MNF usually is a benign localized condition, but can be as extensive as till the ora, giving a pseudoappearance of leukocoria and masquerade conditions like retinoblastoma or retinopathy of prematurity. A thorough dilated fundus examination is a must in all such cases, preferably under anesthesia, to prevent misdiagnosis and mistreatment. As long as the retrolental membrane is neither in the visual axis nor causing any coexisting traction, observation is warranted in such cases.

## Notes

### Competing interests

The authors declare that they have no competing interests.

### Informed consent

The patient has viewed the content and images of this case report and has consented to the submission of the case report for publication.
